# 25 Years after Vi Typhoid Vaccine Efficacy Study, Typhoid Affects Significant Number of Population in Nepal

**DOI:** 10.1371/journal.pone.0077974

**Published:** 2014-01-06

**Authors:** Deepak Bajracharya, M. Imran Khan, Alfred Pach, Parisha Shrestha, Nilesh Joshi, Shyam R. Upreti, Thomas Wierzba, Mahesh Puri, Sushant Sahastrabuddhe, R. Leon Ochiai

**Affiliations:** 1 MITRA Samaj Pani Pokhari, Kathmandu, Nepal; 2 Group for Technical Assistance, Sanepa, Nepal; 3 International Vaccine Institute, Seoul, South Korea; 4 Child Health Division, Department of Health Services, Ministry of Health and Population, Kathmandu, Nepal; Menzies School of Health Research, Australia

## Abstract

*Salmonella* Typhi, first isolated in 1884, results in infection of the intestines and can end in death and disability. Due to serious adverse events post vaccination, whole cell killed vaccines have been replaced with new generation vaccines. The efficacy of Vi polysaccharide (ViPS) vaccine, a new generation, single-dose intramuscular typhoid vaccine was assessed in Nepal in 1987. However, despite the availability of ViPS vaccine for more than 25 years, Nepal has one of the highest incidence of typhoid fever. Therefore we collected information from hospitals in the Kathmandu Valley from over the past five years. There were 9901 enteric fever cases between January 2008 and July 2012. 1,881 of these were confirmed typhoid cases from five hospitals in the Kathmandu district. Approximately 70% of the cases involved children under 15 years old. 1281 cases were confirmed as *S.* Paratyphi. Vaccines should be prioritized for control of typhoid in conjunction with improved water and sanitation conditions in Nepal and in endemic countries of Asia and Africa.

## Introduction

It has been almost 150 years since Eberth first identified *Salmonella* Typhi (*S.* Typhi) for the first time; one of the very first bacteria to be isolated at the time [1]. In spite of advances in preventive medicine, humans continue to suffer from typhoid and paratyphoid fever. The symptoms of typhoid fever range from very mild cases of fever treated in outpatient clinics to severe forms that result in intestinal perforation and hospitalization [2]. The current global burden estimates indicate that more than 90% of typhoid fever cases occur in countries in South and South East Asia [3]. Yet, the epidemiology and burden of typhoid fever are still not well understood [4].

Typhoid fever presents challenges that range across its diagnosis, treatment and prevention [6]. The most effective interventions are improvements in water and sanitation, which have resulted in the reduction of typhoid fever incidence in developed nations where typhoid fever was once a major cause of death [5]. Developments in water and sanitation in these settings occurred in parallel to improvements in other areas, such as health education and economic advancements. Similar methods of control and eradication of the disease in many countries of Asia demand considerable long-term socio-behavioral and economic progress and resources which are scarce in these countries [6]. Thus, an interim preventive measure such as vaccination is critical in these settings. There is evidence from around the world that the disease has been successfully controlled through vaccination and infrastructure development [7], [8]. Yet, the use of available and effective vaccines has not received the attention and support needed for controlling typhoid fever in low and middle income countries. Moreover, the growing rate of multi-drug resistant strains of *S.* Typhi in such settings has resulted in the increased cost of treatment and the difficulty in managing the severity of symptoms [2]. There are currently two licensed vaccines for typhoid, Ty21a and Vi polysaccharide vaccine. Ty21a is an oral live attenuated vaccine available in two formulations (enteric coated capsule and a liquid formulation). Three doses of Ty21a in enteric-coated capsules conferred 67% efficacy over three years of follow-up and 62% protection over seven years of follow-up given on an every other day schedule [9]. Currently only the enteric-coated capsule formulation is manufactured. Ty21a is not WHO prequalified- limiting its use through United Nations procurement system such as UNICEF, the major donor of vaccines in developing countries.

Twenty five years ago The New England Journal of Medicine published the efficacy results of a clinical trial conducted in Nepal using the Vi polysaccharide (ViPS) vaccine, one of the two licensed typhoid vaccines [10]. ViPS vaccine is single dose and can be stored at +2 to +8 degrees Celsius. The ViPS vaccine from one manufacturer is WHO prequalified and hence could be purchased in countries where vaccine supply is supported by UNICEF [11]. The vaccine has been used effectively in successful programs in a number of developing countries [12], [13] as well as in travel clinics in wealthy and developed nations. In spite of the evidence for effectiveness of Vi polysaccharide vaccine, the vaccine has not been used routinely in Nepal, a country with one of the highest rates of endemic typhoid globally. Despite the absence of robust surveillance data, there are reports of significant typhoid fever in the country [14].

Here, we present data from hospitals in the Kathmandu districts, one of the three in the valley, demonstrating higher numbers of blood culture-confirmed typhoid fever cases. Our data should elicit joint efforts from national and international organizations to control typhoid fever using existing vaccines in conjunction with other preventive efforts to save significant health, social and economic costs to the population in Nepal, who contributed to the knowledge of typhoid vaccines more than two decades ago. The use of typhoid vaccines is an important example of obstacles and challenges faced before a licensed and effective vaccine is made available to the people who need it the most [15].

## Methods

This 5-year retrospective hospital based data report is part of an ongoing study in Nepal. This study examines the effectiveness of the ViPS vaccine and its feasibility in public health programs as part of advocacy efforts aimed at using existing vaccines for the control of typhoid fever in Nepal. Data from blood culture confirmed cases of typhoid fever were collected from hospitals providing inpatient and outpatient services in the Kathmandu District, Nepal. Out of 37 hospitals, 11 hospitals provided data on enteric fever. The remaining hospitals did not have either laboratory facilities for establishing proper blood culture confirmation or a regular information recording system for fever cases. Surveillance staff visited the hospitals with a support letter from the Child Health Division of the Ministry of Health and Population and extracted 2008–2012 data from outpatients and laboratory log books on: all fever cases reported, total blood culture assessments performed on fever cases, total blood culture positives, total enteric fever cases, and total typhoid cases identified.

Data was available from hospitals where records of patients were maintained. Where possible, we also collected information on the age of the cases presenting to these hospitals. The age categories of cases were divided into less than 5 years, 5–15 years and more than 15 years. We also collected data, where available, for each month of the year since 2008. We collected summary data from each facility and did not collect information on patient identification. Data was entered in Microsoft excel and analyzed in STATA version 11.1.

### Ethical considerations

The study was approved by Department of Child Health, Ministry of Health and Population, government of Nepal. There was no direct contact of the study staff with the patients and data was collected retrospectively and information collected from the hospitals did not contain any identifiers of the patients. A written or verbal informed consent was waived by Ministry of Health and Population.

## Results

In the last five years, 30,586 febrile cases presented to at least five hospitals in Kathmandu districts (Table 1). A total of 9,901 blood culture proven enteric fever cases was recorded in the 11 hospitals in the Valley (Table 2). The annual number of cases ranged from a minimum of 947 in 2008 to a maximum of 3010 in 2011. Janamaitri Hospital reported the majority of cases, with 3,463 (35%) since 2008 (Table 2). There were 1881 cases of blood culture confirmed typhoid cases reported from five hospitals during the same time period. There is more than threefold increase in typhoid fever cases with 165 cases in 2008 to 770 cases in 2011 (Table 3). While typhoid fever is present throughout the year, the majority of cases are clustered around the summer months, from March to July. Among 3,857 cases for which the patient age was available, 456 (12%), 1,155 (30%), 2,246 (58%) cases were from younger than five years, between five and equal to fifteen years and older than fifteen years, respectively (Table 4).

**Table 1:** Distribution of enteric fever by year comparing with overall fever cases.

S.No.	Description	2008		2009		2010		2011		2012	
1	Total Number presented with fever	4474		5762		7303		9466		3581	
2	Total number presented with fever and blood culture done	3967	89%*	4849	84%	6529	89%	8562	90%	3190	89%
3	Total number presented with fever, blood culture done and positive growth (Typhi and paratyphi)	259	6%	382	7%	1052	14%	1091	12%	149	4%
4	Total number presented with fever, blood culture done and *S.* Typhi	165	4%	237	4%	515	7%	757	8%	67	2%
5	Total number presented with fever blood culture done and *S.* Paratyphi	94	2%	177	3%	569	8%	340	4%	106	3%

**Note: data comes from four Hospitals – (AH, HAMS, KMC, KMH)**.

percentages are based on number of febrile patients presenting to the hospitals.

**Table 2:** Annual distribution of enteric fever in Kathmandu, Nepal.

	2008	2009	2010	2011	2012*	Total
Tribhuvan University Teaching Hospital	161	364	80	70	35	710
STIDH	204	165	284	189	75	917
IF Children Hospital	240	228	53	129	73	723
Janamaitri Hospital	0	809	934	1293	427	3463
Army Hospital	125	168	322	269	129	1013
Kathmandu Model Hospital	50	175	690	782	20	1717
Kathmandu Medical College	46	38	37	36	24	181
HAMS	38	33	35	33	0	139
OM Hospital	4	63	193	32	43	331
Nepal Police Hospital	64	87	120	98	70	439
Bir Hospital	19	73	59	79	38	268
Total	947	2203	2807	3010	934	9901

data for full year of 2012 was not available at the time of data collection.

**Table 3:** Annual distribution of typhoid fever cases by hospitals in Kathmandu Valley, Nepal.

	2008	2009	2010	2011	2012*	Total
Army Hospital	72	82	122	105	30	411
Kathmandu Model Hospital	35	105	345	625	13	1123
Kathmandu Medical College	40	33	33	11	24	141
HAMS	18	17	15	16	0	66
OM Hospital	0	31	81	13	15	140
Total	**165**	**268**	**596**	**770**	**82**	**1881**

Data for 2012 is until October.

**Table 4:** Age distribution of enteric fever cases annually in Kathmandu Valley, Nepal.

	Total *Enteric fever cases*	<5 years		5–15 years		15 years	
		*N*	*%*	*n*	*%*	*n*	*%*
2008	631	105	17	258	41	268	42
2009	1328	142	11	315	24	871	66
2010	746	81	11	259	35	406	54
2011	805	77	10	220	27	508	63
2012	347	51	15	103	30	193	56
**Total**	3857	456		1155		2246	

Note: Hospitals in above data: TH, IFCH, KMC, HHC, OH, and NPH.

TH- Tribhuvan University Teaching Hospital.

IFCH- International Friendship's Children Hospital.

KMC- Kathmandu Medical College.

HHC- Helping Hands Community Hospital.

OH- Om Hospital.

NPH- Nepal Police Hospital.

AH- Army Hospital.

HAMS- Hospital for Advanced Medicine and Surgery.

## Discussion

The data from the reporting hospitals clearly indicates that enteric fever is a common infectious disease affecting all age groups in Kathmandu Valley and Nepal, though adolescents and adults are at the greatest risk. Typhoid and Paratyphoid fever are significant infectious diseases in the population in the valley. In the hospitals where information on fever and blood culture were available, 4% to 8% of patients presenting with fever were typhoid and 2% to 4% were paratyphoid fever. These numbers are underestimates considering the sensitivity of blood culture technique. In addition we were unable to collect uniform information from all of the 37 hospitals that cater to infectious diseases in the valley.

The population density in Kathmandu Valley has increased due to migration from rural Nepal. This has developed a pressure on water and hygiene infrastructure, resulting in compromised conditions and the spread of infectious diseases [16]. Retrospective data from various sources suggest that typhoid fever peaked in the valley due to the civil unrest occurring outside of the valley in 2001, which led to a large, susceptible population moving into the valley [17]. Other studies from the Kathmandu Valley have reported high rates of first line anti-microbial resistance to S. Typhi [14], [18]. Antimicrobial resistance not only adds to the cost and difficulty of treatment, but also prolongs the duration of treatment and may result in unwanted complications such as gut perforation [19]. Despite the emergence of drug resistance, availability of antimicrobials has markedly reduced typhoid fever related mortality, a major initiator for government supported efforts for setting priorities for the control of diseases. We were unable to capture information on typhoid mortality due to a lack of typhoid specific mortality surveillance.

Vaccination against typhoid fever alone is not sufficient for the full control of the disease [7]. Instead, typhoid fever control demands a comprehensive strategy that includes prevention of the disease through hand washing, maintaining personal and environmental hygiene, adequate sewage treatment and availability of safe and clean drinking water to reduce problems of water-borne infections and the spread of enteric fever. In addition, as the world has witnessed a decline in mortality due to availability of antimicrobials, access to timely and appropriate clinical care also helps in reducing complication rates due to typhoid and the costs related to typhoid fever morbidity. The cost of the ViPS vaccine (9 USD per dose), that it can only be given to older than 2 years population, challenges of delivery in routine public health programs; and the widespread availability and use of antibiotics are the reasons why government policy has not been developed for typhoid vaccination. Moreover, long-term, infrastructure development strategies are necessary for curbing the spread of diseases, as well as for maintaining the general health of the population. The data on the effectiveness of ViPS vaccine provides a strong rationale for its use among other strategies in meeting public health prevention needs for controlling typhoid fever and is relevant for the control of disease in the Kathmandu Valley.

It is quite poignant and ironic that, while the ViPS vaccine was tested and licensed based on data from Nepal in 1987, the country has not had the opportunity to use the vaccine in its immunization program [10]. Unfortunately, the general public has not been well informed about the use of the vaccine for preventing typhoid fever. In addition, the cost of the locally available vaccine is prohibitively expensive for most of the Nepali population. To address this issue, a pilot typhoid vaccination project was conducted in the Kathmandu Valley between 2010 and 2011. The results show a high population demand for the prevention of the disease through the use of vaccines. Following the pilot typhoid vaccination project, there has been increased interest in and commitment from the Ministry of Health and Population and District Public Health Offices for introducing the ViPS vaccine in high risk populations.

Government officials closely follow World Health Organization (WHO) recommendations for the use of vaccines and adhere to its procurement guidelines, that is, pre-qualification standards for the use and purchase of vaccines. The existing WHO pre-qualified ViPS vaccine is presently too expensive for developing country governments with their existing health budget. However, typhoid fever is one of the priority diseases for the Global Alliance for Vaccines and Immunization (GAVI), which, in its meeting in September 2011, committed its support for conjugate typhoid fever vaccines. A conjugate vaccine has an increased duration of efficacy and may be used for children under two years old. There are conjugate typhoid fever vaccines in the pipeline, two of them are licensed for use in India. The WHO-prequalification of these vaccines is still a few years away. Considering the burden of typhoid fever in the Kathmandu Valley and in other parts of South Asia, it is imperative to consider ways to support the use of existing vaccines. Unquestionably, local public health leadership should play a vital role in addressing the typhoid control agenda, but international experts and agencies have a strong influence in ensuring access and support of the use of existing typhoid vaccines in countries such as Nepal [20]. Access is a key word for which international agencies need to assume accountability in order to address the unmet public health needs of lower income communities that continue to suffer from typhoid in spite of having hosted trials that resulted in the licensure and use of such vaccines in wealthy populations who can afford it.

### Limitation

The data presented is hospital-based and thus may not represent the full picture of the distribution and rates of typhoid fever in the Valley and Nepal. We were also unable to collect information from all hospitals of Kathmandu district, limiting the estimation of typhoid fever burden. The inconsistency of information collection in these hospitals limited our ability to compare data between hospitals and calculate proportion for different indicators. Two hospitals from the list of hospitals that provided us information on *S.* Typhi isolation rates had significantly higher numbers compared to other hospitals. It is likely that many of cases captured in the hospitals do not live in Kathmandu valley.

Our data also does not consider blood culture sensitivity. However, the cases of typhoid fever presented here are likely only the tip of the iceberg with the actual incidence being possibly much higher. There are other studies from the Kathmandu Valley and from other parts of Nepal which indicate the pervasiveness of typhoid fever in the country [14]. Data for the later quarter of 2012 was not available at the time this manuscript was written.
